# Preterm Birth International Collaborative Australasia Branch: Expert Consensus on Diagnosis and Treatment of Neonatal Lactose Intolerance (2025)

**DOI:** 10.1002/pdi3.70044

**Published:** 2026-03-27

**Authors:** Zhengli Wang, Shuping Han, Xinzhu Lin, Hui Wu, Lingfen Xu, Jun Tang, Wei Hao, Chao Yu, Xiaomei Tong, Juan Zhang, Rong Ju, Xiaoli Xie, Long Chen, Fang Li, Xingwang Zhu, Liping Xu, Lanlan Geng, Wei Zhou, Xueyun Ren, Chuanfeng Li, Guoping Chen, Wenyan Tang, Jiang Duan, Zhi Zhao, Tao Pan, Zhongliang Li, Yingli Wu, Siqi Zhuang, Ying Ouyang, Falin Xu, Zheng Chen, Yiyang Liu, Zhoujie Peng, Lei Bao, Yu He, Lin Kong, Xianlan Zheng, Lijuan Xu, Qiao Shen, Qian Zhang, Junli Li, Junjun Wang, Yuan Shi

**Affiliations:** ^1^ Children’s Hospital of Chongqing Medical University Chongqing China; ^2^ Women’s Hospital of Nanjing Medical University/Nanjing Women and Children’s Healthcare Hospital Nanjing Jiangsu China; ^3^ School of Medicine Women and Children’s Hospital Xiamen University Xiamen Fujian China; ^4^ First Hospital of Jilin University Changchun Jilin China; ^5^ Shengjing Hospital of China Medical University Shenyang Liaoning China; ^6^ West China Second University Hospital Sichuan University Chengdu Sichuan China; ^7^ Shandong Provincial Hospital Affiliated to Shandong First Medical University Jinan Shandong China; ^8^ The First Affiliated Hospital of Chongqing Medical and Pharmaceutical College Chongqing China; ^9^ Peking University Third Hospital Beijing China; ^10^ School of Medicine Chengdu Women’s and Children’s Central Hospital University of Electronic Science and Technology of China Chengdu Sichuan China; ^11^ Women and Children’s Hospital of Chongqing Medical University/Chongqing Health Center for Women and Children Chongqing China; ^12^ Bishan District Health Center for Women and Children of Chongqing Chongqing China; ^13^ Zhangzhou Affiliated Hospital of Fujian medical University Zhangzhou Fujian China; ^14^ Guangzhou Women and Children’s Medical Center Guangzhou Guangdong China; ^15^ Affiliated Hospital of Jining Medical University Jining Shandong China; ^16^ Qujing Maternal and Child Health‐Care Hospital Qujing Yunnan China; ^17^ The First Affiliated Hospital of Harbin Medical University Harbin Heilongjiang China; ^18^ Jiangxi Maternal and Child Health Hospital/Jiangxi Children’s Medical Center Nanchang Jiangxi China; ^19^ The First Affiliated Hospital of Kunming Medical University Kunming Yunnan China; ^20^ Shaanxi Provincial People’s Hospital Xi’an Shaanxi China; ^21^ Children’s Hospital Affiliated to Soochow University Soochow Jiangsu China; ^22^ WeiFang Maternal and Child Health Hospital WeiFang Shandong China; ^23^ The Seventh Affiliated Hospital of Sun Yat‐sen University Shenzhen Guangdong China; ^24^ Sun Yat‐sen Memorial Hospital Sun Yat‐sen University Guangzhou Guangdong China; ^25^ The Third Affiliated Hospital of Zhengzhou University Zhengzhou Henan China; ^26^ Children’s Hospital Zhejiang University School of Medicine Hangzhou Zhejiang China; ^27^ School of Clinical Medicine Shandong Second Medical University Weifang Shandong China; ^28^ Chongqing University Three Gorges Hospital Chongqing China

**Keywords:** diagnosis, lactose intolerance, lactose malabsorption, newborn, preterm, treatment

## Abstract

This consensus aims to standardize the diagnosis and management of neonatal lactose intolerance (LI). A multidisciplinary working group was established under the auspices of the Preterm Birth International Collaborative (Australasia Branch). The development process included a comprehensive literature review of databases, which identified 61 publications. Recommendations were formulated using the Grading of Recommendations, Assessment, Development and Evaluation system for evidence evaluation. A modified Delphi method was used to achieve consensus. The consensus establishes that neonatal LI presents with nonspecific gastrointestinal symptoms, requiring stool assessment for diagnosis. Screening tests include fecal reducing sugar or urinary galactose tests, and the diagnosis is confirmed through elimination testing while excluding mimics. Treatment prioritizes lactase‐supplemented breastfeeding; formula‐fed infants receive lactase or reduced‐lactose formulas. Probiotics with β‐galactosidase activity can be used as an adjuvant therapy, and evidence‐based lactase preparations are recommended, particularly for preterm infants. Treatment duration should be individualized, with a general minimum of 2 weeks; however, the routine use of lactose‐free formula to prevent LI in preterm infants is not recommended. This consensus provides practical evidence‐based guidance to standardize the diagnosis and management of neonatal LI, aiming to reduce variation in practice and improve patient outcomes. It emphasizes the importance of maintaining breastfeeding while incorporating lactase supplementation and highlights the need for further high‐quality research, particularly randomized controlled trials, to refine diagnostic criteria and therapeutic strategies for preterm and very preterm infants. The recommendations are intended to support clinicians in making informed decisions while promoting family‐centered care.

**Trial Registration:**

International Practice Guidelines Registry Platform (PREPARE‐2024CN606)

## 1 Introduction

Lactose intolerance (LI) is a clinical syndrome characterized by uncomfortable symptoms in patients with lactose malabsorption (LM) following the ingestion of lactose‐containing foods [1]. It is classified, based on the etiology of lactase deficiency, as congenital, primary, developmental, or secondary. Congenital LI is an extremely rare genetic disorder. Congenital lactase deficiency induces intractable diarrhea in neonates after they ingest human milk or lactose‐containing formula, precipitating life‐threatening dehydration with severe metabolic disturbances and requiring lactose‐free feeding. Primary LI is caused by relative or absolute lactase deficiency due to single nucleotide polymorphisms in the transcriptional promoter region of the lactase gene, with symptoms generally manifesting in childhood [1, 2]. Developmental LI, particularly in preterm infants, occurs because of immature digestive system development, causing temporarily insufficient lactase activity or secretion in the intestine and consequent LM and LI symptoms. Secondary LI stems from transient suppression of lactase production or decrease in lactase activity following gut mucosal damage caused by intestinal pathologies, trauma, or systemic factors, inducing dual LM and clinical manifestations [2, 3]. Neonatal LI is mainly developmental or secondary, with diverse and nonspecific clinical manifestations. This condition is determined by multiple factors, such as lactose intake, individual lactase levels, co‐ingested foods, gut transit speed, and microbiome profile [3]. It shares nonspecific symptoms with diverse gastrointestinal disorders. The true prevalence of neonatal LI remains obscured by diagnostic limitations, including age‐specific method scarcity, demanding sampling protocols, and institutional auxiliary test scarcity. The epidemiology of neonatal LI in China has only been reported in small‐sample studies: existing data indicate that the incidence of LI in neonates born in obstetric departments is approximately 2.7%–5.8% [4, 5], while in hospitalized neonates the incidence ranges from 18.4% to 19.7% [6].

To standardize the diagnosis and treatment of neonatal LI, the National Clinical Research Center for Child Health and Disorders (Chongqing) established a Consensus Development Working Group in April 2024. The group included experts in neonatal and pediatric medicine, nursing, and healthcare insurance, as well as parent representatives. Through comprehensive evidence retrieval, appraisal, and integration, and after multiple rounds of expert review and discussion, the Chinese Expert Consensus on Diagnosis and Treatment of Neonatal Lactose Intolerance (2025) (hereinafter referred to as “this Consensus”) was formulated. Addressing four common clinical issues, the group developed 10 recommendations aimed at standardizing the diagnosis and treatment of neonatal LI.

## 2 Methods for Developing the Consensus

### 2.1 Target Population

#### 2.1.1 Target Patients

Neonates and preterm infants with a corrected gestational age < 44 weeks.

#### 2.1.2 Target Users

Pediatricians, neonatologists, child health physicians, dietitians, perinatal healthcare workers, community healthcare workers, and related nursing staff.

### 2.2 Applicable Settings

Medical institutions at all levels engaged in neonatal diagnosis and treatment.

### 2.3 Development Process

A multidisciplinary expert working group was established to develop this consensus, comprising 32 neonatology specialists, 3 pediatric gastroenterology experts, 2 nurses, and 2 clinical nutritionists. In addition, input was sought from 2 parents of neonates with LI and 1 healthcare insurance expert. All participants signed written conflict‐of‐interest statements. Through consensus meetings, the group refined the key clinical issues concerning the diagnosis and treatment of neonatal LI.

Relevant literature was retrieved from PubMed, Web of Science, Embase, Cochrane Library, the Chinese Medical Knowledge Database, China National Knowledge Infrastructure, Wanfang Database, and the Chinese Biomedical Literature Database, covering publications from their inception to June 30, 2024. Search terms included neonate, term infant, preterm infant, low birth weight infant, small for gestational age infant, extremely low birth weight infant, extremely preterm infant, very low birth weight infant, lactase, LI, lactase deficiency, and LM. Keywords were supplemented with synonyms and related terms adapted to the requirements of each database. Following additional retrieval based on expert panel discussions, a total of 61 studies were included: 4 guidelines, 7 expert consensuses or standards, 2 meta‐analyses/systematic reviews, 15 randomized controlled trials (RCTs), 16 observational studies, 2 retrospective studies, and 15 reviews. These studies formed the basis for recommendations, which were formulated by considering multiple dimensions: quality of evidence, balance of benefits and harms, parental preferences and values, and resource availability. Following the Grading of Recommendations, Assessment, Development and Evaluation (GRADE) system [7], management strategies were systematically summarized, and evidence‐based recommendations were derived. This process allowed a transparent appraisal of the evidence supporting each recommendation, as illustrated in Table 1. In scenarios with scarce or low‐quality evidence, the expert panel employed a modified Delphi method, voting until consensus was achieved.

**TABLE 1:** Representations of quality of evidence and strength of recommendations.

Certainty of evidence	
High quality	A
Moderate quality	B
Low quality	C
Very low quality	D
Strength of recommendation	
Strong recommendation for using intervention	1
Conditional recommendation for using intervention	2
Good practice statement	GPS

Abbreviation: GPS, Recommendation based on consensus experience.

### 2.4 Manuscript Development and External Review

The consensus project was initiated on April 12, 2024. Registration was completed, and the consensus expert panel was established on May 3, 2024. Collection of clinical questions concluded on June 10, 2024, followed by the completion of literature retrieval on July 2, 2024 and evidence synthesis on October 18, 2024. The first draft was finalized on January 7, 2025. After multiple rounds of deliberation, revisions by the working group, and supplementary literature retrieval to incorporate critical references, the manuscript was submitted following final revisions on July 20, 2025.

### 2.5 Dissemination and Update

This consensus is disseminated through the journal *P* *ediatric* *D* *iscovery*. It will be systematically updated approximately every 5 years in response to evolving evidence and policy changes.

## 3 Recommendations

### 3.1 Identification of Symptoms of Neonatal LI

Recommendation 1: The clinical manifestations of neonatal LI are diverse and non‐specific, often presenting intestinal colic, abdominal distension, hyperactive bowel sounds, diarrhea, regurgitation, and irritability. Changes in stool characteristics and an acidic stool odor should be identified promptly to facilitate further diagnosis and treatment (C1).

The clinical manifestations of LI include gastrointestinal and systemic symptoms [8], although neonates primarily present with gastrointestinal signs. A recent retrospective study of 113 infants with LI within the first six months of life reported that abdominal distension, intestinal colic, diarrhea, nausea, and irritability were common, with more than 50% experiencing neonatal‐onset symptoms (56.7%). Moreover, 56.6% of infants had stool consistency between solid and liquid, and 30.1% showed blood streaks in the stool, although neonatal‐specific data were not described separately [9]. A prospective observational study by Huan et al. [10], which included hospitalized neonates, found that among 154 neonates with lactase deficiency, 42 (27.3%) were diagnosed with LI. Common symptoms included hyperactive bowel sounds after feeding (76.2%, 32/42), vomiting (71.4%, 30/42), and diarrhea (66.7%, 28/42). Affected neonates may also exhibit crying and restlessness after feeding and before defecation, showing improvement after bowel movements. Stool is often yellow or blue–green, egg‐like or loose‐paste in consistency, may contain milk curds or foam, and frequently emit an acidic odor [4, 11, 12, 13]. Clinical manifestations differ between preterm and term infants; however, most studies focus on term or near‐term neonates. A multicenter prospective randomized controlled trial by Zha et al. [14] involving preterm infants with LI (gestational age ≤ 34 weeks) found that 56.1% (87/155) experienced abdominal distension, 21.9% (34/155) had vomiting, and only few reported diarrhea (median bowel movements: 2.0 times/day).

A recent multicenter survey of physicians from specialized neonatal outpatient clinics reported that over 50% observed a predominance of preterm infants with LI, presenting with diarrhea, regurgitation or vomiting, abdominal distension, and unexplained crying [15]. During the neonatal period, rapid growth and high nutrient demands make infants particularly vulnerable. Diarrhea caused by LI can damage the intestinal mucosa, exacerbating symptoms and leading to persistence. Consequences may include unexplained crying, perianal skin irritation, impaired weight gain, and reduced calcium and phosphorus absorption [1, 3, 12, 16]. These factors underscore the importance of early recognition and identification of neonatal LI.

### 3.2 Diagnosis and Differential Diagnosis of Neonatal LI

Recommendation 2: For symptomatic neonates, preliminary screening options include fecal reducing sugar test or urinary galactose assay (C1).

Recommendation 3: For symptomatic neonates, clinical diagnosis can be made through diagnostic elimination test while actively ruling out other diseases (GPS).

The National Institutes of Health (NIH) consensus defines LI as “gastrointestinal symptoms occurring in individuals with lactose malabsorption after ingesting a single dose of lactose in a blinded provocation test, with symptoms absent during placebo ingestion [17]. Blinded multi‐dose provocation tests can clarify lactose digestion and tolerance, determining individualized tolerance thresholds. However, such tests are difficult to perform in neonates. Direct and indirect detection methods include jejunal mucosal biopsy, genetic testing, oral lactose tolerance test, hydrogen breath test, and fecal pH measurement.

1. Fecal reducing sugar test: Lactase deficiency allows unhydrolyzed lactose to reach the colon, with partial excretion in the feces. Fecal reducing sugar reflects lactose decomposition. This test is convenient, rapid [18, 19], and commonly used for neonatal LI screening in Chinese hospitals [15]. Because fecal sugar is primarily in fecal fluid, false negatives may occur if the fluid is absorbed by diapers or if the sample is stored too long, as bacteria can degrade sugars.
2. Urinary galactose assay: Lactose is hydrolyzed into galactose by lactase, which is predominantly excreted in urine. Lactase activity can thus be indirectly assessed by measuring urinary galactose. A negative result indicates absent lactase activity, suggesting LI. This simple and widely used method provides rapid clinical guidance [20, 21, 22, 23]. However, neonates, particularly preterm infants, often have developmental LI with residual lactase activity, which can yield detectable galactose and potential false negatives.
3. Diagnostic elimination test: In China, the diagnostic capability for neonatal LI is limited, with low specificity and sensitivity, and routine screening is not implemented in many hospitals [15]. Clinical diagnosis is typically based on the appearance of LI symptoms following ingestion of standard lactose‐containing milk, provided other pathologies are excluded. Key diagnostic indicators include rapid symptom resolution upon lactase supplementation or low‐/zero‐lactose formula introduction, with symptom recurrence upon lactase cessation or resumption of regular lactose feeds [24]. This is consistent with the “blinded provocation test” approach for confirming LI in the NIH consensus [17].
4. Other detection methods: Despite its methodological simplicity and operational convenience, fecal pH testing demonstrates suboptimal sensitivity and specificity for neonatal LI diagnosis, rendering its diagnostic utility limited in this population [14, 21, 25]. Jejunal mucosal biopsy is costly, invasive, and technically challenging [11]; genetic testing is mainly used to predict LM and lactase nonpersistence (LNP) and shows significant regional variations [26]; oral lactose tolerance test necessitates blood glucose monitoring at predetermined intervals following standardized lactose administration, entailing invasive sampling procedures [3, 11]; and the hydrogen breath test exhibits suboptimal time efficiency and parental compliance. Additionally, detectable breath hydrogen levels are observed in healthy asymptomatic neonates and preterm populations [27, 28, 29, 30]. With these limitations considered, these methods are not suitable for preliminary screening of neonates with LI.

Recommendation 4: Neonatal LI predominantly manifests as gastrointestinal symptoms, necessitating differential diagnosis from necrotizing enterocolitis, cow's milk protein allergy, infectious diarrhea, gastrointestinal malformations, gastroesophageal reflux, and feeding intolerance (GPS).

Neonatal LI predominantly presents with gastrointestinal symptoms and requires differential diagnosis from necrotizing enterocolitis, cow's milk protein allergy, infectious diarrhea, gastrointestinal malformations, antibiotic‐associated diarrhea, gastroesophageal reflux, and feeding intolerance, often co‐occurring or sequentially manifesting. These conditions often overlap or coexist. Beyond fecal reducing sugar test or urinary galactose assay, differentiation can be conducted based on clinical symptoms and other auxiliary examinations.

1. Neonatal necrotizing enterocolitis (NEC): Neonates with necrotizing enterocolitis exhibit severe systemic symptoms, including reduced activity, recurrent vomiting, pallor, shock, gross bloody stools, and peritonitis. In contrast, neonates with LI are typically in good condition, and gross bloody stools are rarely observed. Intestinal dilatation, fixed bowel loops, pneumatosis intestinalis, portal venous gas, ascites, or pneumoperitoneum may be noted in radiologic findings in NEC, whereas no such imaging abnormalities are observed in LI. Laboratory findings in NEC often include neutropenia, thrombocytopenia, and elevated nonspecific inflammatory markers [31], which are absent in LI.
2. Cow's milk protein allergy (CMPA): Neonatal CMPA, the most common neonatal food allergy, is an immune response to specific milk proteins. Its digestive symptoms resemble LI, and the two conditions may coexist [24]. CMPA may also present with extra‐digestive signs such as atopic dermatitis or eczema. Differentiation can be aided by diagnostic elimination tests, fecal reducing sugar, or urinary galactose assays. When symptoms cannot be explained by a single condition, the possibility of coexistence should be considered [32, 33].
3. Infectious diarrhea: Infectious diarrhea in neonates is typically caused by viral or bacterial intestinal infections and may occur with or without vomiting, hyperactive bowel sounds, or fever. Stool is often watery, purulent, or blood‐streaked. Infectious diarrhea can also develop secondary to neonatal LI. Auxiliary examinations may reveal abnormal white blood cell counts, elevated C‐reactive protein or procalcitonin, numerous white or red blood cells in stool, or positive bacterial culture or viral isolation [33, 34].
4. Gastroesophageal reflux and congenital gastrointestinal malformations: Gastroesophageal reflux, congenital megacolon, intussusception, intestinal volvulus, short bowel syndrome, and meconium ileus can present with vomiting, abdominal distension, abnormal bowel sounds, abdominal discomfort, and diarrhea. Preliminary differentiation from neonatal LI can be performed using abdominal X‐rays and gastrointestinal ultrasound. If conventional treatment is ineffective and organic or severe diseases are excluded, a diagnostic elimination test for LI can be performed for differentiation.
5. Feeding intolerance: Feeding intolerance is typically assessed using gastric residual volume, abdominal distension, vomiting, or feeding outcome indicators, despite the lack of internationally standardized diagnostic criteria. In China, it is diagnosed when “gastric residual volume exceeds 50% of the preceding feeding volume with concomitant vomiting and/or abdominal distension” or when “enteral nutrition protocols fail, including reduction, delay, or interruption of feeding” [35]. Clinical symptoms of feeding intolerance often overlap with those of LI, and the two conditions may coexist. Differentiation can be achieved using diagnostic elimination tests, fecal reducing sugar tests, or urinary galactose assay.

### 3.3 Treatment of Neonatal LI

Lactose metabolites enhance intestinal microecology, stimulate peristalsis, improve calcium and phosphorus absorption, and support neurodevelopment [36, 37, 38]. Lactase is localized on the apical surface of intestinal villi, rendering it susceptible to secondary deficiency following enteric mucosal injury. Lactase deficiency is particularly common in preterm infants; for example, enzyme activity in neonates born at 34 weeks of gestation is approximately 30% of that in term neonates at birth [39]. Active intervention is not always required in neonates—particularly term infants—with very mild symptoms because of the self‐limiting nature of LI. However, treatment should be initiated in neonates presenting with severe LI symptoms (e.g., infantile colic, abdominal distension, hyperactive bowel sounds, diarrhea, regurgitation, and irritability) or significant complications affecting feeding, sleep, growth, or perianal skin integrity. Management of neonatal LI primarily combines lactose restriction or lactase supplementation with symptomatic therapy. This dual approach alleviates symptoms and prevents complications. Once significant symptom resolution and lactase recovery are achieved, interventions should be withdrawn gradually to restore normal lactose exposure [3, 40].

Recommendation 5: For breastfed neonates with LI, continued breastfeeding combined with lactase preparations is the recommended first‐line treatment (A1).

Recommendation 6: For neonates receiving standard lactose‐containing formulas, initial intervention entails either lactase supplementation or transition to reduced‐lactose formulations (B1).

Studies have reported that lactase supplementation in preterm neonates—regardless of feeding modality (breast milk or formula)—is not associated with an increased risk of NEC and demonstrates a favorable safety profile without major adverse effects [16, 41]. A nonrandomized multicenter study by Chen et al. [42], including 117 preterm infants with LI (gestational age ≤ 34 weeks), showed that lactase supplementation effectively treats neonatal LI, promotes weight gain, and does not require discontinuation of breastfeeding. The Expert Consensus on the Use of Breast Milk in Neonatal Intensive Care Units (2021) recommends lactase supplementation for feeding intolerance due to lactase deficiency in preterm infants [43]. These recommendations are consistent with the Expert Consensus on Prevention and Management of Childhood Rotavirus Gastroenteritis (2020 Edition), the Expert Consensus on Enteral Nutrition Management of Preterm Infants (2024), and the Chinese Clinical Practice Guidelines for Acute Infectious Diarrhea in Children [32, 44, 45]. Recommendations for the management of acute diarrhea in non‐malnourished children issued by the Federation of International Societies of Pediatric Gastroenterology, Hepatology, and Nutrition (FISPGHAN) in 2018 stated that infants under six months with acute diarrhea should continue breastfeeding, with lactose‐free formula reserved for diarrhea lasting more than 14 days [34]. Owing to its multiple benefits, breastfeeding is strongly promoted. Discontinuing breastfeeding as the initial treatment for LI in breastfed neonates is not recommended in current guidelines; instead, lactase supplementation is advised to alleviate symptoms [2, 8].

Studies indicate that adding lactase preparations [14, 16, 41, 42, 46] or using lactose‐free formula mitigates symptoms in neonates with LI who are fed lactose‐containing formula [47]. Intestinal colic is closely associated with LI [48]; feeding with lactose‐free, low‐lactose or partially hydrolyzed formulas can effectively reduce infantile colic, vomiting, and frequency of bowel movements [49, 50].

Lactose‐free formula is one of the common therapeutic modalities for LI; however, its long‐term use may pose the risk of abnormal calcium absorption, specifically in preterm infants [51]. Switching to lactose‐free formula can temporarily relieve LI symptoms; however, avoiding lactose is not conducive to inducing endogenous lactase activity and secretion in neonates [52, 53], and formula transition is required after significant alleviation of symptoms. Chen et al. [42] reported that preterm infants with LI who received breast milk/preterm formula supplemented with lactase preparations exhibited better weight gain than those fed lactose‐free extensively hydrolyzed formula or amino acid formula. A case‐control investigation by Li et al. [54] found superior growth parameters and nutritional indicators in lactase‐supplemented preterm neonates with LI compared with those receiving lactose‐free formulations. Therefore, the routine use of lactose‐free formula as initial treatment is not recommended; instead, adding lactase preparations or switching to low‐lactose formula should be attempted first. For neonates on mixed feeding who develop LI, the intervention can involve adding lactase preparations during breastfeeding and either adding lactase preparations or switching to a low‐lactose formula during feeding with normal lactose‐containing formula. In addition, if LI symptoms do not improve after initial intervention, a short‐term lactose‐free formula is warranted post‐differential diagnosis, with stepwise reintroduction of standard formula upon symptom resolution.

Recommendation 7: Individualize neonatal LI treatment duration based on clinical symptom trajectory while maintaining a general therapy duration of ≥ 2 weeks (B1).

Three prospective RCTs [14, 42, 46] in Chinese preterm neonates (gestational age ≤ 34 weeks) with lactose maldigestion demonstrated partial symptom resolution after adjunct lactase therapy for 7 days. After 14 days, LI symptoms were significantly alleviated relative to controls, with post‐intervention corrected gestational age (CGA) reaching a mean of 33–34 weeks. Kien et al. reported that lactose decomposition by colonic flora in neonates (gestational age 30–32 weeks) at 14 days postnatal can largely compensate for lactase insufficiency [55]. A double‐blind RCT by Erasmus et al. [41] in preterm infants (gestational age ≤ 34 weeks) showed that weight gain and serum albumin levels were significantly higher in the lactase dry powder intervention group than in the control group after 2 weeks of therapy. However, no additional clinical benefits were observed when lactase supplementation continued beyond a mean CGA of 34 weeks. Intestinal mucosal injury from various factors can lead to secondary lactase deficiency [3], and it typically takes several weeks to months for the intestine to recover sufficient lactase [1]. Special cases, such as infants with short bowel syndrome, may require longer intervention [56]. The severity of developmental and secondary lactase deficiency [2] varies; thus, treatment duration should be individualized based on the trajectory of clinical symptoms. Evidence synthesis suggests treatment for ≥ 2 weeks for developmental/secondary LI, aligning with intestinal recovery timelines. Notably, prolonging the intervention duration does not increase clinical benefits but instead increases medical costs and the economic burden on parents [41]. For optimized therapeutic precision, neonates with LI whose symptoms significantly improve after 34 weeks CGA should undergo a gradual reduction in intervention through dynamic monitoring, balancing efficacy against overtreatment.

Recommendation 8: Routine use of lactose‐free formula for preventing LI in preterm infants is not recommended (B1).

Although lactase deficiency is highly prevalent in preterm neonates, the incidence of clinically manifest LI remains comparatively low. Contributing factors include early feeding, which promotes lactase activity [52, 53], and lactose decomposition by colonic flora, which compensates for malabsorption caused by lactase insufficiency [55, 57]. In addition, LI symptoms are experienced only when lactase activity falls below 50% [58]. A small‐sample study reported that even in extremely preterm infants, intestinal lactose hydrolysis efficiency exceeds 98% within 5 days of initiating feeding [59]. Compared with preterm formula, lactose‐free formula has lower caloric content because of the absence of lactose, as well as reduced levels of fat, protein, and trace elements. Routine preventive use of lactose‐free formula significantly increases the risk of malnutrition and adverse effects on neurological development [51]. Both the Clinical Guidelines for the Diagnosis and Treatment of Feeding Intolerance in Preterm Infants (2020) and the Expert Consensus on Enteral Nutrition Management in Preterm Infants with Special Conditions (2024) in China do not recommend routine use of lactose‐free or low‐lactose formulas or lactase preparations for the prevention of feeding intolerance [35, 60].

Recommendation 9: Probiotics with β‐galactosidase activity can be used as an adjuvant therapy for neonatal LI (C2).

Emerging evidence suggests that promoting intestinal colonization with β‐galactosidase–producing probiotics may enhance lactose digestion, representing a potential adjunctive strategy for managing LI [37]. Strains that express β‐galactosidase include *Bifidobacterium lactis, Lactobacillus acidophilus, Lactobacillus brevis, Lactobacillus casei, Lactobacillus plantarum, Lactobacillus rhamnosus, Lactobacillus salivarius, Lactococcus lactis, Streptococcus thermophilus*, and others [8]. In Chinese published systematic reviews [37, 61] and the expert consensus on lactose intolerance and scientific milk consumption [62], the therapeutic benefits of probiotic supplementation were underscored, revealing improvement in the clinical symptoms of LI. However, these studies were limited to older children and adults, with no dedicated research on neonates. Concerns remain regarding potential risks of probiotic use in preterm infants, particularly those who are extremely or very preterm, including systemic infection, excessive immune activation, transfer of antibiotic resistance genes, and harmful metabolic activity. Therefore, probiotic use in preterm neonates warrants caution, given heterogeneous clinical trajectories and the potential for life‐threatening adverse events. High‐quality studies are needed to fully evaluate the safety and efficacy of probiotics in this population.

### 3.4 Safety and Efficacy of Lactase Intervention

Recommendation 10: It is recommended to use evidence‐validated lactase preparations to treat neonatal LI, specifically in preterm infants (GPS).

**FIGURE 1:**
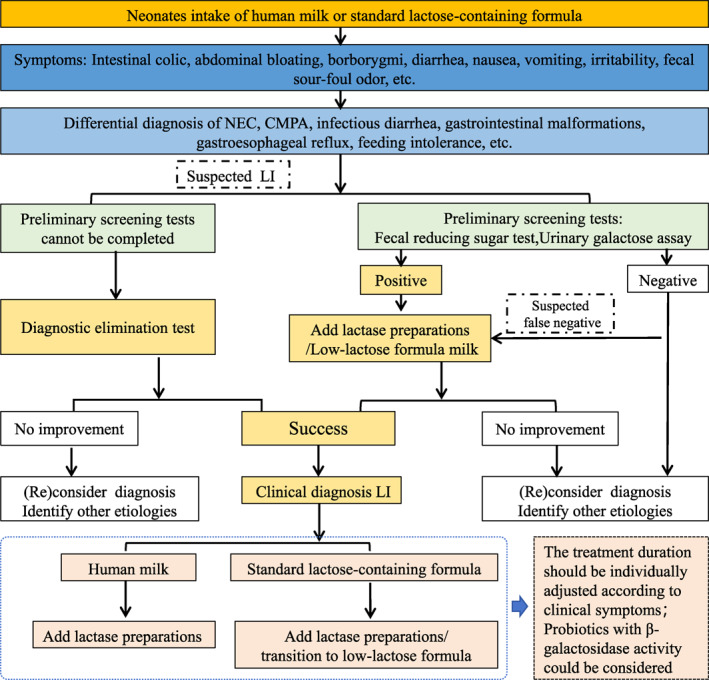
Flowchart for diagnosis and treatment of lactose intolerance in neonates. CMPA, cow's milk protein allergy; LI, lactose intolerance; NEC, necrotizing enterocolitis.

Patient safety remains a critical concern in clinical practice. Although specialized infant formulas are subject to stringent regulatory standards, lactase enzyme preparations are classified as food or dietary supplements in China. Consequently, their development, manufacturing, and distribution are not subjects to the rigorous oversight applied to pharmaceuticals. Multiple studies [14, 41, 42, 46, 54] have shown that lactase enzyme preparations added to breast milk or infant formula for preterm infants are well‐tolerated, with no significant adverse effects reported. These preparations effectively alleviate LI‐associated clinical symptoms in preterm infants, reduce the need for formula switching, and help lower parental anxiety [14, 42, 46]. However, neonates—particularly preterm infants—have immature physiological systems, requiring heightened safety considerations for ingested foods or medications. Various lactase enzyme preparations are commercially available; however, many lack robust, evidence‐based data supporting their safety and efficacy in neonates, particularly preterm infants. The unique vulnerabilities of this population imply that the clinical selection of lactase enzyme preparations for LI treatment is strongly recommended, provided their safety and efficacy have been proven. The flowchart for the diagnosis and treatment of LI in neonates is shown in Figure 1.

## 4 Limitations

The quality of evidence provided by the research reports referenced in this consensus is suboptimal, with a particular paucity of large‐scale RCTs or systematic reviews. Consequently, most of the recommendations formulated in this consensus are of low grade. Future large‐sample RCTs are therefore needed to establish the optimal intervention timing for neonatal LI and to evaluate the clinical utility of preventive interventions in extremely or very preterm cohorts. Additionally, probiotic interventions for neonatal LI require further RCT validation; essential research should focus on estimating the effects of the optimal initiation time, dosage, duration, and composition of probiotic preparations on relevant clinical endpoints.

## 5 Statement

The core content of this article is derived from the *Expert Consensus on the Diagnosis and Management* *of Neonatal Lactose Intolerance (2025)* [63], originally published in Chinese in the *Chinese Journal of Neonatology*. In the absence of a dedicated expert consensus on neonatal LI, we have summarized the evidence‐based clinical experience from China into this expert consensus and now present it in English through *Pediatric Discovery*. This publication aims to share Chinese clinical insights and standardized recommendations with the global neonatal medical community, providing a valuable reference for the diagnosis, treatment, and management of neonatal lactose intolerance globally.

Lead Writers: Wang Zhengli, Han Shuping, Lin Xinzhu, Wu Hui, Xu Lingfen, Tang Jun, Hao Wei, Yu Chao, Shi Yuan *

Consensus Expert Group: Peking University Third Hospital, Beijing, China (Xiaomei Tong, Juan Zhang); Chengdu Women's and Children's Central Hospital, School of Medicine, University of Electronic Science and Technology of China (Rong Ju, Xiaoli Xie); Women and Children's Hospital of Chongqing Medical University/Chongqing Health Center for Women and Children, Chongqing, China (Long Chen, Fang Li); Bishan District Health Center for Women and Children of Chongqing, Chongqing, China (Xingwang Zhu); Children's Hospital of Chongqing Medical University, Chongqing, China (Lei Bao, Yu He, Lin Kong, Yuan Shi, Zhengli Wang, Xianlan Zheng, Lijuan Xu, Qiao Shen); The First Affiliated Hospital of Chongqing Medical and Pharmaceutical College, Chongqing, China (Chao Yu); Zhangzhou Affiliated Hospital of Fujian medical University, Fujian, China (Liping Xu); Guangzhou Women and Children's Medical Center, Guangdong, China (Lanlan Geng, Wei Zhou); Affiliated Hospital of Jining Medical University, Jining, China (Xueyun Ren); Qujing Maternal and Child Health‐care Hospital, Yunnan, China (Chuanfeng Li); The First Affiliated Hospital of Harbin Medical University, Heilongjiang, China (Guoping Chen); First Hospital of Jilin University, Jilin, China (Hui Wu); Jiangxi Maternal and Child Health Hospital/Jiangxi Children's Medical Center, Jiangxi, China (Wenyan Tang); The First Affiliated Hospital of Kunming Medical University, Yunnan, China (Jiang Duan); Women's Hospital of Nanjing Medical University/Nanjing Women and Children's Healthcare Hospital, Jiangsu, China (Shuping Han); Shandong Provincial Hospital Affiliated to Shandong First Medical University, Shandong, China (Wei Hao); Shaanxi Provincial People's Hospital, Shaanxi Xi'an, China (Zhi Zhao); West China Second University Hospital, Sichuan University, Sichuan, China (Jun Tang); Children's Hospital Affiliated to Soochow University, Jiangsu (Tao Pan); WeiFang Maternal And Child Health Hospital, Shandong, China (Zhongliang Li, Yingli Wu); Women and Children's Hospital, School of Medicine, Xiamen University, Fujian, China (Xinzhu Lin); The Seventh Affiliated Hospital of Sun Yat‐sen University, Guangdong, China (Siqi Zhuang); Sun Yat‐sen Memorial Hospital, Sun Yat‐sen University, Guangdong, China (Ying Ouyang); The Third Affiliated Hospital of Zhengzhou University, Henan, China (Falin Xu); Children's Hospital, Zhejiang University School of Medicine, Zhejiang, China (Zheng Chen); Shengjing Hospital of China Medical University, Liaoning, China (Lingfen Xu).

Methodological Guidance for Literature Search: Children's Hospital of Chongqing Medical University, Chongqing, China (Jungang Zhao); Norwich Medical School, University of East Anglia, Norwich, UK (Fujian Song).

Secretariat and Evidence Evaluation Group: Children's Hospital of Chongqing Medical University, Chongqing, China (Zhengli Wang, Qiao Shen, Lijuan Xu, Qian Zhang, Junli Li); The First Affiliated Hospital of Chongqing Medical and Pharmaceutical College, Chongqing, China (Chao Yu); Shandong Provincial Hospital Affiliated to Shandong First Medical University, Jinan, China (Wei Hao); School of Clinical Medicine, Shandong Second Medical University, Weifang, China (Yiyang Liu); The Third Affiliated Hospital of Chongqing University, Chongqing, China (Zhoujie Peng).

Parent representatives of lactose‐intolerant neonates: Man Feng, Mei Zhou.

Medical Insurance Expert: Children's Hospital of Chongqing Medical University, Chongqing, China (Junjun Wang).

## Author Contributions

Yuan Shi contributed to the overall conceptualization and design of this consensus. He guided and critically reviewed the literature retrieval strategy, undertook the revision and refinement of the entire manuscript, and was responsible for quality control throughout the drafting process. Zhengli Wang, Shuping Han, Xinzhu Lin, Hui Wu, Lingfen Xu, Jun Tang, Wei Hao and Chao Yu participated in consensus discussions and the development of recommendations, and were responsible for drafting the initial consensus manuscript and finalizing the revised version. Zhengli Wang, Qiao Shen, Lijuan Xu, Qian Zhang, Junli Li, Chao Yu, Wei Hao, Yiyang Liu and Zhoujie Peng conducted literature searches, performed evidence evaluation, and completed evidence synthesis. Junjun Wang provided professional consultation on medical insurance and related economic policies. All other experts participated in consensus discussions and the formulation of recommendations, and provided professional academic input and support.

## Funding

National Key Research and Development Program of China (Grant 2022YFC2704805); Key Research and Development Program of Jiangxi Province (Grant 20243BBI91020); Jiangxi Provincial Natural Science Foundation (Grant 20242BAB23079); Peak Discipline Initiative of Children's Hospital of Chongqing Medical University (Grant CHCMU‐2024‐XKDF‐1002).

## Conflicts of Interest

Yuan Shi and Yu He are members of Pediatric Discovery Editorial Board. To minimize bias, they were excluded from all editorial decision‐making related to the acceptance of this article for publication. The other authors declare no conflicts of interest.

## Data Availability

Data sharing not applicable to this article as no datasets were generated or analysed during the current study.
